# Clinical Manifestations of Supra-Large Range Nonperfusion Area in Diabetic Retinopathy

**DOI:** 10.1155/2022/8775641

**Published:** 2022-02-03

**Authors:** Nianting Tong, Liangyu Wang, Huimin Gong, Lin Pan, Fuxiang Yuan, Zhanyu Zhou

**Affiliations:** ^1^Department of Ophthalmology, Qingdao Municipal Hospital, Qingdao, China; ^2^Dalian Medical University, Dalian, China

## Abstract

**Objective:**

We describe the clinical manifestations of supra-large range nonperfusion area (SLRNPA) in diabetic retinopathy (DR).

**Methods:**

This was a retrospective case-control study. A total of 260 eyes of 236 patients with DR who underwent pars plana vitrectomy in the Department of Ophthalmology of Qingdao Municipal Hospital from February 2016 to June 2019 were enrolled. Fundus fluorescein angiography was performed after surgery to determine whether SLRNPA or non-SLRNPA in DR was present. All demographic and clinical data were carefully collected.

**Results:**

Forty-one eyes of 22 patients were diagnosed with SLRNPA in DR (15.77% of all eyes). Compared to non-SLRNPA, SLRNPA patients were more likely to be male and younger with earlier DR onset, a smoking history, other comorbidities, and a higher HbA1c level. SLRNPA in DR eyes exhibited more neovascular glaucoma (NVG) and diabetic keratopathy (DK) than did other eyes. Such eyes were more likely to require anti-VEGF therapy before surgery or a silicone oil or a gas tamponade during surgery and to suffer from persistent corneal epithelial erosion and NVG recurrence after surgery.

**Conclusions:**

SLRNPA in DR is a severe status of DR. Treatment for DR patients with SLRNPA is difficult, and the prognosis is poor, so clinicians must thus pay more attention to SLRNPA in DR.

## 1. Introduction

Diabetes mellitus (DM) is a global epidemic. According to the World Health Organization (WHO), the global DM number will increase to about 592 million by 2035 [1, 2]. Especially in recent decades, China's economy has developed rapidly, but this has increased the prevalence of overweight and obesity, and thus, the level of diabetes [3, 4]. Diabetic retinopathy (DR) is associated with visible clinical changes in the retinal fundus caused by diabetes of long duration and poor control of blood glucose levels. Although good systemic control of glucose levels delays DR onset and progression, DR affects almost all diabetics at some time, even if hyperglycemia is well-controlled [5–7]. The primary lesions of DR include retinal nonperfusion areas (NPAs) developing secondary to retinal capillary closure [8]. The appearance of NPAs precedes neovascularization and triggers a need for interventional treatments such as photocoagulation. In our clinic, we found that on fundus fluorescein angiography (FFA), some patients lacked retinal capillary perfusion beyond the posterior pole of the fundus. We also found that the treatment of such patients was extraordinarily difficult, being often accompanied by the recurrence of neovascular glaucoma (NVG) and persistent corneal epithelial erosion (PCEE) during follow-up. Upon careful search of the literature, no relevant reports were found. Given the FFA manifestations, we termed this status of DR as supra-large range nonperfusion area (SLRNPA). We suggest that SLRNPA may be a severe status of DR with difficult treatment and poor prognosis. Here, we describe this severe status of DR, compare it to the other status of DR, and investigate its characteristics. We seek to establish a theoretical basis for early prevention, diagnosis, and treatment.

## 2. Materials and Methods

### 2.1. Study Design and Participants

This was a retrospective case-control study. Proliferative DR (PDR) patients who underwent pars plana vitrectomy (PPV) due to the vitreous hemorrhage and were followed-up in the Department of Ophthalmology of Qingdao Municipal Hospital from February 2016 to June 2019 were included. The study adhered to the relevant tenets of the Declaration of Helsinki and was approved by the Ethics Committee of Qingdao Municipal Hospital, Qingdao, China. Informed consent was obtained from all patients or their guardians prior to surgery and FFA. All medical records and patient demographics were retrospectively reviewed after approval was received from the Institutional Review Board of Qingdao Municipal Hospital. All patients underwent a full ophthalmological examination, including measurement of best-corrected visual acuity (BCVA) (logMAR) and intraocular pressure (IOP), slit-lamp examination, and fundus examination (using a binocular indirect ophthalmoscope after pupil dilation).

### 2.2. Image Acquisition

To distinguish SLRNPA from DR, FFA was performed using the Spectralis Heidelberg Engineering system. After standard intravenous injection of 5 mL 10% (w/v) fluorescein sodium (Alcon Laboratories Inc.), FFA images were obtained over a 55° area 45 s and 2 and 10 min later (the early, middle, and late phases of angiography, respectively). Central images were centered on the macula; we also obtained superior, temporal, nasal, and inferior images to allow clear visualization of the peripheral edges of the retinal vasculature.

### 2.3. Grading

**Figure 1:**
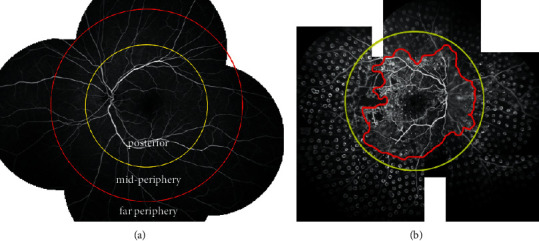
(a) On a typical image, the retina is divided into three sections (from the posterior pole to the periphery). The posterior pole is the area within the yellow circle with the fovea at the center and a radius of 10 mm, and the midperipheral retina is the area between the yellow and red circles; the latter has the fovea as its center and a radius of 15 mm. The peripheral retina comprises the area beyond the red circle. (b) Typical FFA image of an eye with SLRNPA in a DR patient; no retinal capillary perfusion is evident beyond the posterior pole of the fundus.

Two experienced graders (NT and LW) who met the requisite quality-assurance standards assessed all FFA images in terms of the retinal perfusion areas and NPAs evident. Following Silva et al. [9], the extent and distribution of retinal capillary perfusion were scored in the following three circles centered on the fovea (such that radii were measured from the fovea): posterior section (radius less than 10 mm), midperipheral section (radius 10–15 mm), and far peripheral section (radius more than 15 mm). If the perfusion area lay only within the posterior section, the graders diagnosed a case of SLRNPA; non-SLRNPA was diagnosed otherwise. If the graders disagreed, an advanced grader (ZZ) made the final decision.  Figure 1(a) shows how the retina was divided into three areas in a healthy retina, and Figure 1(b) shows a typical FFA image of an SLRNPA eye in DR.

### 2.4. Data Collection

All medical records and patient demographics were reviewed. We collected data on sex, age, smoking and alcohol consumption histories, comorbidities (hypertension, hyperlipidemia, and coronary heart disease), diabetes onset time and course, and the HbA1c level. We recorded the data before, during, and after surgery. The axial length, need for retinal photocoagulation, NVG and diabetic keratopathy (DK) status, anti-VEGF treatment history, and lens status were recorded preoperatively. Information on the points of retinal photocoagulation, intraocular tamponade use, and surgery duration was collected perioperatively. PCEE occurrence and NVG recurrence were recorded postoperatively.

### 2.5. Statistical Analysis

SPSS version 17.0 (SPSS, Chicago, IL, USA) was used to analyze all the data. Descriptive results are given as the means ± standard deviations and were compared using the paired *t*-test (quantitative variables). Categorical variables are given as counts with percentages and were compared using the chi-squared test.

## 3. Results

We enrolled 260 eyes from 236 patients (139 men and 107 women) of mean age 56.44 ± 6.26 years (range 35–71 years). All had type 2 diabetes. The average age at disease onset was 43.82 ± 5.13 years (range 32–62 years), and the mean duration of diabetes was 12.62 ± 4.07 years (range 2–23 years).

For all patients, the mean HbA1c level was 7.98 ± 1.06%, and 51.69% had chronic medical conditions (hypertension, hyperlipidemia, and/or coronary heart disease). About 33.47% of the patients had a history of smoking and 36.44% a history of alcohol consumption. After FFA grading, all patients were divided into SLRNPA and non-SLRNPA groups. A total of 41 eyes of 22 patients were categorized into the SLRNPA group (only posterior retinal perfusion was evident) and 219 eyes of 214 patients the non-SLRNPA group.

Table 1 compares the two groups. They differed significantly in terms of sex, age, smoking history, comorbidity status, DR onset time, and HbA1c level but not in terms of alcohol consumption history or disease course. For all eyes listed in Table 2, the presurgery incidences of NVG, DK, and anti-VEGF treatments were significantly higher in the SLRNPA group than in the non-SLRNPA group. The intraocular tamponade placement rate and surgical duration were slightly greater in the SLRNPA group. The incidences of PCEE occurrence and NVG recurrence after surgery were significantly higher in the SLRNPA group. No significant between-group difference was found in terms of the retinal photocoagulation requirement, lens status, axial length before surgery, or number of pan-retinal photocoagulation (PRP) points during surgery.

We next describe two representative cases with SLRNPA. In both, during treatment and follow-up, PCEE developed and NVG recurred.

### 3.1. Case 1

**Figure 2:**
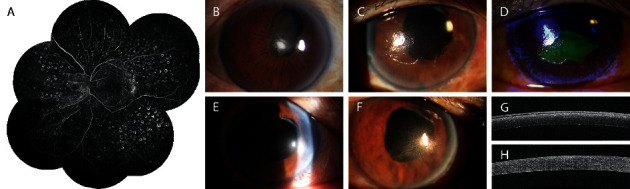
(a) FFA image of the left eye of Case 1. No retinal capillary perfusion is evident beyond the posterior pole of the fundus. This is an eye with SLRNPA in a patient with DR. (b) Anterior segment image taken on the first visit, showing partial posterior iris adhesion, neovascularization of the iris surface, and a cataract. (c) Image of a large-scale defect of the corneal epithelium. (d) The cornea is fluorescein-positive (via slit-lamp microscopy) at 3 months after surgery. The epithelial defect was treated via placement of a bandage contact lens (e), and the eye subsequently recovered (f). (g, h) Anterior segment optical coherence tomography images taken before (g) and after (h) bandage contact lens treatment. After treatment, the adhesion of the corneal epithelium to the stroma was no longer loose (g) but tight instead (h).

A 63-year-old Chinese male complained of gradual vision loss in the left eye of about 3 months in duration. His BCVA was finger-counting, and the intraocular pressure was 34 mmHg. Ophthalmic examination revealed a cataract, NVG (Figure 2(b)), and vitreous hemorrhage. He underwent phacoemulsification combined with intraocular lens implantation and PPV with PRP. At 1 week after surgery, he underwent FFA (Figure 2(a)). As the left eye retinal perfusion area was confined to the posterior section, we diagnosed SLRNPA. At about 3 months after surgery, a large-scale defect of the corneal epithelium was found on slit-lamp microscopy (Figures 2(c) and 2(d)). A bandage contact lens was placed to aid the recovery of the corneal epithelium (Figure 2(e)). The defect recovered but with residual corneal clouding (Figure 2(f)).

### 3.2. Case 2

**Figure 3:**
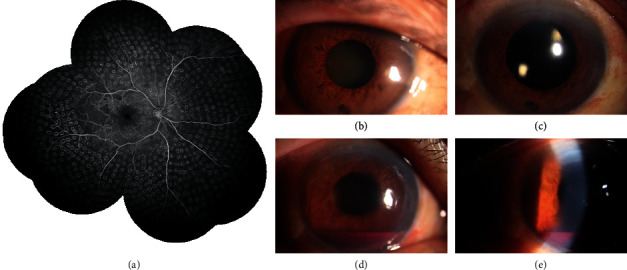
(a) FFA image of the right eye of Case 2. All retinal capillary perfusion lies within the posterior pole of the fundus. This is another eye with SLRNPA in a DR patient. (b) Anterior segment image taken on the first visit, showing neovascularization of the iris surface and a cataract. (c) Image of the anterior segment at 1 week after surgery, showing regression of iris surface neovascularization. (d, e) Images of the anterior segment at 1 month after surgery, showing recovery from NVG and hyphema.

A 57-year-old Chinese male complained of sudden visual loss of the right eye of about 3 days in duration. His BCVA was hand motion, and the intraocular pressure was 13 mmHg. Ophthalmic examination revealed a cataract, NVG, and serious vitreous hemorrhage; he underwent phacoemulsification combined with intraocular lens implantation and PPV with PRP. At 1 week after surgery, he underwent FFA (Figure 3(a)); the right eye was diagnosed with SLRNPA (Figure 3(a)). The NVG and hyphema recovered about 1 month later (Figures 3(d) and 3(e)).

## 4. Discussion

DR is one of the most serious microvascular complications of diabetes. In a previous study [10], a significant (inverse) association was found between the perfusion index (PI) and DR severity, suggesting that the PI may serve as a useful biomarker when diagnosing DR and assessing progression. Another group used ultrawide field fluorescein angiography to assess the relationship between peripheral nonperfusion and DR severity [9]. They found that the retinal NPA and nonperfusion index (NPI) correlated highly with predominantly peripheral lesions (PPLs) and DR severity. As both the presence and extent of PPLs were associated with an increased risk of DR progression, clinical PPLs may closely reflect the extent of nonperfusion and ischemia, explaining the increased risk of progression. In the present study, we focused on DR with perfusion only confined to the posterior section. Given the importance of peripheral retinal perfusion, the complete loss thereof in SLRNPA eyes in DR indicates that this particular status of DR might be associated with more severe, rapid progression, and poor prognosis.

### 4.1. NVG Occurrence and Recurrence in SLRNPA DR Eyes

We found that NVG development prior to surgery and NVG recurrence after surgery were significantly more common in SLRNPA eyes than in other eyes in DR. NVG is a secondary form of glaucoma generally associated with poor visual prognosis. The underlying pathogenesis of NVG is posterior segment ischemia, which most commonly occurs secondary to PDR or retinal vein occlusion (RVO) [11]. As an important indicator of progression from non-PDR to PDR, the development of retinal capillary NPAs means that hyperglycemia has caused the retina to deteriorate to the proliferative state. Neovascularization was found to correlate positively with an increasing proportion of capillary nonperfusion (the ischemic index) within the area of occlusion [12], as such, NVG in DR patients reflects retinal vascular occlusion [13]. Retinal nonperfusion thresholds indicating retinal neovascularization, the distribution and area of retinal nonperfusion in eyes with severe non-PDR, PDR, neovascularization of the optic disc, and retinal neovascularization elsewhere have been established [14]. The cited authors measured the total area of retinal nonperfusion, area of posterior pole retinal nonperfusion, and area of peripheral retinal nonperfusion and found that eyes with at least 107.3 disc areas of nonperfusion were at risk of PDR. As mentioned above, the peripheral retinal NPA is associated with DR progression. A prospective interventional study of patients with treatment-naïve PDR showed that, at all times, the area of peripheral capillary nonperfusion was significantly greater in patients with active PDR at 6 months after PRP treatment [15]. In other words, the area of peripheral capillary nonperfusion may serve as a predictive marker for PDR activity after treatment with PRP. In SLRNPA eyes, perfusion was found only in the posterior area, indicating that ischemia was very severe (i.e., peripheral retinal perfusion was totally lost). Such severe ischemia, especially in the retinal midperipheral and peripheral areas, triggered significantly more NVG prior to surgery and more NVG recurrence after surgery in SLRNPA eyes than in other DR eyes.

### 4.2. DK and PCEE

DK is an ocular complication developing secondarily to diabetes [16]. Diabetics may exhibit many subclinical corneal abnormalities, including reduced epithelial barrier function [17], abnormalities in the shapes of epithelial and endothelial cells [18], basement membrane thickening [19], and decreased corneal sensation [20]. These pathological changes cause DK, the clinical manifestations of which include PCEE, superficial punctate keratopathy, delayed epithelial regeneration, and decreased corneal sensitivity that may compromise visual acuity or trigger permanent loss of vision [21]. No study has yet investigated the relationship between the range and distribution of retinal NPAs and DK severity, but many studies have reported that corneal sensitivity may serve as a potential marker of diabetic neuropathy [22] that correlates positively with the extent of retinal ischemia. Here, we found significantly higher incidences of DK and PCEE recurrence after surgery in SLRNPA eyes than in other DR eyes, possibly reflecting the much more severe fundus ischemia and higher HbA1c levels in such patients. Poorly controlled blood glucose levels trigger decreases in corneal sensation and epithelial barrier function, abnormalities in the shapes of epithelial and endothelial cells, basement membrane thickening, and decreased adhesion between the corneal epithelium and stroma. Eventually, DK and PCEE develop.

### 4.3. Demographics: Age, Sex, Comorbidities, Time of Onset, and HbA1c Level

In our present study, patients in the SLRNPA group were younger than patients in the non-SLRNPA group; the disease had commenced earlier, and the mean HbA1c level was higher. As SLRNPA is a novel clinical entity, we cannot compare our work to that of others. As mentioned above, a high NVG incidence or recurrence rate is a characteristic of eyes with SLRNPA in DR. We examined risk factors for NVG and found that our results were compatible with those of other reports. A retrospective multicenter study focused on the risk factors for NVG after 25-gauge vitrectomy to treat PDR with vitreous hemorrhage and found that younger age, uncontrolled diabetes (a high HbA1c level), no PRP, and postoperative vitreous hemorrhage were risk factors for postvitrectomy NVG [23]. Younger patients who underwent vitrectomy to treat PDR exhibited more severe anatomical issues at the time of vitrectomy than older patients and experienced a poorer anatomical success rate (higher rates of postoperative complications including NVG and rapid progression of retinal neovascularization) [24]. Indicators of poor diabetes control are associated with a higher risk of DR [25]. Control of the blood glucose level reduces the risks of retinopathy development and progression [26]. A higher HbA1c level (reflecting poorer diabetic control) was evident in our SLRNPA group compared to the non-SLRNPA group. We found that the DM courses of the patients with SLRNPA and non-SLRNPA were comparable, but other studies have reported that the mean diabetic duration in NVG patients is shorter than that in non-NVG patients. Notably, we found that DM onset was significantly earlier in patients in the SLRNPA group, which may explain why the patients in this group were younger.

Our work had several limitations. This was a retrospective case-control study and only Chinese DR patients with type 2 diabetes were enrolled. The FA images were also captured using an ordinary lens (55°). Although FFA images were obtained superiorly, temporally, nasally, and inferiorly (to allow clear visualization of the peripheral edge of the retinal vasculature), some peripheral retinal images were missing, and the images were not as good as those yielded by ultrawide field angiography.

In summary, FFA revealed that eyes with SLRNPA are characterized by the lack of capillary perfusion beyond the posterior section of the retina. In terms of demographic features, DR patients with SLRNPA were more likely to be male, younger at disease onset, and have a history of smoking, comorbidities, and higher HbA1c levels compared to other DR patients with non-SLRNPA.

SLRNPA in DR is a severe status of DR given the higher incidences of NVG and DK before surgery, greater need for intraocular tamponade during surgery, and higher probabilities of PCEE and NVG recurrence after surgery. This condition thus requires more attention in the clinic and might predict more aggressive treatment, such as rigorous PRP treatment, regular anti-VEGF intravitreal injections to prevent NVG, and worse prognosis, such as higher occurrence and recurrence of NVG and PCEE.

## Figures and Tables

**Table 1:** Comparison of patients with SLRNPA or non-SLRNPA in different groups.

		SLRNPA (*n* = 22)	Non-SLRNPA (*n* = 214)	*p* value
Sex	Male/female	17/5	122/102	0.025
Age	Years	52.14 ± 6.87	56.88 ± 6.04	0.001
Smoking status	Yes/no	12/10	67/147	0.028
Alcohol consumption	Yes/no	11/11	75/139	0.165
Comorbidity	Yes/no	17/5	105/109	0.011
	0/1/2/3	5/5/4/8	109/49/30/26	0.009
Onset time	Years	40.00 ± 4.95	44.22 ± 5.00	0.001
Course (minus age at onset)	Years	12.14 ± 3.45	12.67 ± 4.13	0.560
HbA1c	>9/≤9	11/11	25/189	≤0.001
		9.09 ± 1.27	7.86 ± 0.96	≤0.001

Comorbidities include hypertension, hyperlipidemia, and coronary heart disease. 0: no comorbidity; 1: one comorbidity; 2: two comorbidities; 3: all three comorbidities. SLRNPA: supra-large range nonperfusion area; HbA1c: glycated hemoglobin.

**Table 2:** Comparison of eyes with SLRNPA or non-SLRNPA in different groups.

		SLRNPA eyes (*n* = 41)	Non-SLRNPA eyes (*n* = 219)	*p* value
RP	Yes/no	17/24	140/79	0.512
NVG	Yes/no	22/19	39/180	≤0.001
DK	Yes/no	13/28	35/184	0.017
Lens status	Phakia	29	144	0.104
	Pseudophakia	10	73	
	Aphakia	2	2	
Anti-VEGF injection	Yes/no	29/12	71/148	≤0.001
Axial length	mm	23.66 ± 0.95	23.89 ± 0.92	0.152
PRP	Points	1.494.07 ± 240.79	1,452.05 ± 258.07	0.335
Tamponade	Yes/no	15/26	41/178	0.011
Duration	Min	133.68 ± 39.01	124.19 ± 26.47	0.054
PCEE	Yes/no	15/26	17/202	≤0.001
Recurrence of NVG	Yes/no	17/5	8/31	≤0.001

RP: retinal photocoagulation; PRP: panretinal photocoagulation; NVG: neovascular glaucoma; DK: diabetic keratopathy; PCEE: persistent corneal epithelial erosion.

## Data Availability

The data that support the findings of this study are available on request from the corresponding author. The data are not publicly available because of privacy or ethical restrictions.

## Additional Points

*What Is Already Known about This Topic?* A significant inverse association is apparent between the perfusion index (PI) and diabetic retinopathy (DR) severity, suggesting that the PI may serve as a useful biomarker when evaluating DR and following up on disease progression. The retinal nonperfusion area and nonperfusion index are highly correlated with the existence of (predominantly) peripheral lesions and DR severity. *What Does This Article Add?* Supra-large range nonperfusion area in diabetic retinopathy is characterized by the lack of any capillary perfusion beyond the posterior section of the retina. Eyes with this condition are more likely (than other eyes) to exhibit persistent corneal epithelial erosion and neovascular glaucoma. The authors thus describe a specific status of severe DR.
